# Prediction of Particulate Concentration Based on Correlation Analysis and a Bi-GRU Model

**DOI:** 10.3390/ijerph192013266

**Published:** 2022-10-14

**Authors:** He Xu, Aosheng Zhang, Xin Xu, Peng Li, Yimu Ji

**Affiliations:** 1 School of Computer Science, Nanjing University of Posts and Telecommunications, Nanjing 210023, China; 2 Jiangsu HPC and Intelligent Processing Engineer Research Center, Nanjing 210003, China

**Keywords:** PCCs, correlation, particulate concentration prediction, Bi-GRU

## Abstract

In recent decades, particulate pollution in the air has caused severe health problems. Therefore, it has become a hot research topic to accurately predict particulate concentrations. Particle concentration has a strong spatial–temporal correlation due to pollution transportation between regions, making it important to understand how to utilize these features to predict particulate concentration. In this paper, Pearson Correlation Coefficients (PCCs) are used to compare the particle concentrations at the target site with those at other locations. The models based on bi-directional gated recurrent units (Bi-GRUs) and PCCs are proposed to predict particle concentrations. The proposed model has the advantage of requiring fewer samples and can forecast particulate concentrations in real time within the next six hours. As a final step, several Beijing air quality monitoring stations are tested for pollutant concentrations hourly. Based on the correlation analysis and the proposed prediction model, the prediction error within the first six hours is smaller than those of the other three models. The model can help environmental researchers improve the prediction accuracy of fine particle concentrations and help environmental policymakers implement relevant pollution control policies by providing tools. With the correlation analysis between the target site and adjacent sites, an accurate pollution control decision can be made based on the internal relationship.

## 1. Introduction

In recent decades, China and other developing countries have experienced rapid economic growth and urbanization, and the problems of air pollution have also been inevitable. For example, due to the deterioration of urban particulate pollution closely related to the intensive emission of fine particulate matter (PM_2.5_) and coarse particulate matter (PM_10_), the frequent occurrence of haze weather has attracted worldwide attention [1]. Epidemiological studies have shown that long-term exposure to high concentrations of particulate matter (PM) can lead to serious health risks [2], such as cardiovascular diseases and respiratory diseases. In addition, PM related visibility reduction will also have a negative impact on human production and daily life. Therefore, it is very important to accurately predict the concentration of particulate matter and take countermeasures in advance according to the prediction. However, because the change in particle concentration has a very complex linear relationship which can be affected by many aspects such as space time, wind direction, and humidity [3], it is a very difficult task to accurately predict the change in particle concentration [4].

However, most fine particle concentration prediction models use the time series data of a single station to predict the concentration, and do not take into account the regional correlations among air quality monitoring stations, which will lead to a certain one-sided prediction of fine particle concentration [4,5,6]. There are three types of methods to predict the concentration of particulate concentration. There is one method called the traditional statistical method, such as the AR model, ARIMA model, and GM (1,1) model [5]. Using PM_2.5_ as an example, Zhang et al. analyzed and predicted the change in PM_2.5_ concentration in Fuzhou by using ARIMA model [6]. This method can fit the change trend in the PM_2.5_ concentration and analyze the correlation between PM_2.5_ and meteorological parameters. It is only applicable to small data sets. The second method is the traditional machine learning-based methods, such as normal equation, decision tree, random forest, and other models [7,8]. Hou et al. predicts the PM_2.5_ and PM_10_ concentrations in Beijing by using support vector machine [9], and the prediction results show that it has good generalization ability. This method is more accurate than the statistical prediction model, and can be applied to small and medium-sized data sets, but the effect of long-term prediction of PM_2.5_ concentration is poor. The third method is neural network, such as RNN, LSTM, and GRU [10]. Qadeer et al. predicts the PM_2.5_ hourly concentration of two major cities in South Korea by using the LSTM cyclic neural network. The results show that the model can better predict the PM_2.5_ concentration and is better than iterative decision tree and cyclic neural network methods [11]. This method overcomes the disadvantages of statistics and traditional machine learning methods [12], is suitable for large data sets, and performs well in the long-term prediction of PM_2.5_ concentration [13]. In [14], the authors investigated changes in mass concentrations of particulate matter (PM) during the Coronavirus Disease of 2019 (COVID-19) lockdown. In 2022, Dai et al. carried out research on spatial–temporal characteristics of PM_2.5_ concentrations in China based on multiple sources of data and LUR-GBM during 2016–2021 [15]. All the above research shows the importance of predicting particulate concentration for human society.

In the early stage, the prediction of fine particle concentration is based on empirical subjective judgment, which is not accurate and efficient. The simple linear regression equation is the most commonly used in the traditional model, and the linear fitting is carried out according to the regression principle of minimum absolute error or the penalty minimization principle of least squares loss function. However, linear models can only find the linear relationship between independent variables and dependent variables, but in reality, the relationship between them is often nonlinear, and the traditional methods can only be used to analyze fewer variables and used in smaller data sets, not suitable for big data.

In recent years, many researchers [16,17,18,19] have been using machine learning methods to predict fine particles in the air. Prediction models based on machine learning can generally be divided into two categories, namely, offline models and online models. Offline models can be further divided into two categories, namely, the single model and hybrid model. As long as a single model includes the linear regression model, grey model, Bayesian model, neural network, and artificial intelligence methods. In many studies, linear models such as the autoregressive integrated moving average model (ARIMA) and mixed logistic regression model (MLR) are used to predict the concentrations of PM_2.5_ and PM_10_. When the concentration sequence of fine particles is linear, the prediction results of ARIMA and MLR are more reliable and more explanatory. However, in practice, the change in fine particle concentration is a highly nonlinear, nonstationary, and irregular sequence. The limitation of linear model is that its prediction depends too much on the ability of linear mapping. Compared with linear model, nonlinear model has better prediction effect on extreme concentration. As a typical nonlinear model, artificial intelligence algorithms, such as artificial neural network (ANN), are widely used to predict the concentration of fine particles. However, nonlinear models have their own limitations. For example, they are prone to local optimization and over fitting problems. In recent years, increasingly more researchers have been trying to use variants of the recurrent neural network (RNN), such as the long-term and short-term memory network (LSTM) and gated recurrent neural network, to predict the concentration of fine particles.

The objectives of this work is listed as the following:

1. For the transmission of air pollutants between regions, the concentration of particulate matter has a strong spatial–temporal correlation [20]. The concentration of particulate matter at the target station will be affected by the concentration of pollutants at other stations in the region. Therefore, it is necessary to analyze the correlation between the data for prediction by the machine learning algorithm. In this paper, we proposed a data analysis method based on PCCs (Pearson Correlation Coefficients) [21] to improve the prediction accuracy of particulate concentration.
2. Because the bidirectional LSTM network model can capture the near position information in addition to capturing the key information of the far position compared with the unidirectional LSTM network model, the structure of the gated recurrent neural network model is simpler, and the training of fewer samples can achieve equivalent performance. Therefore, we propose a new model named the bi-directional gated recurrent unit (Bi-GRU) model which can be used in time series prediction.
3. Through the experiments, we verify that the particle concentration prediction model based on PCCs and Bi-GRU can perform real-time particulate concentration forecasting in the coming six hours.

## 2. Theory and Model

### 2.1. Data Sources

In order to evaluate our proposed model, we used the following data sources. A total of 35,064 hourly monitoring data from 12 air quality monitoring stations in Beijing from 1 March 2013 to 28 February 2017 were selected as the required data for the experiments. The Olympic Sports Center station was selected as the target station, and the other 11 stations are the adjacent stations. The longitude and latitude coordinates of these 12 stations are shown in Table 1. 

**Figure 1:**
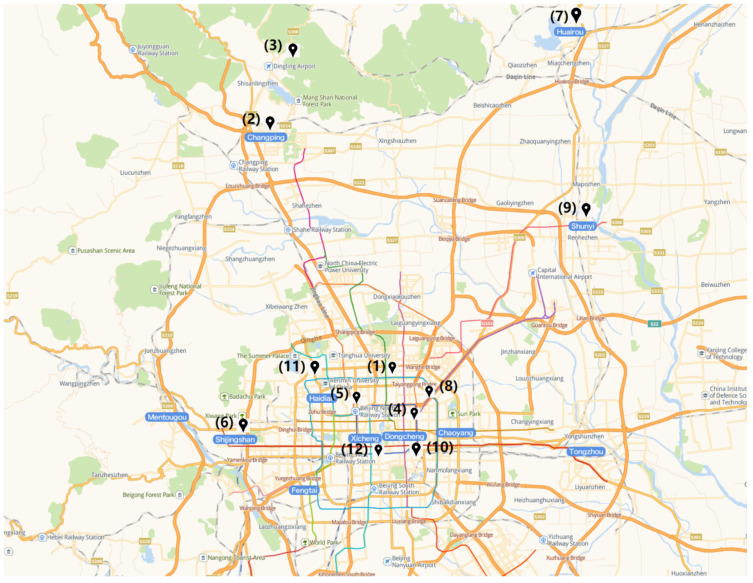
The distribution of stations.

The distribution map is shown in Figure 1 [22].

### 2.2. PCCs

PCCs are a type of coefficient indicating the strength of correlation. They are marked as ***S*** to indicate the degree of correlation between two variables *A* and *B* [23]. The formula is as follows. Where σ*_A_* represents the standard deviation of *A*, σ*_B_* represents the standard deviation of *B*, $\bar{A}$ represents the sample average value of *A*, $\bar{B}$ represents the sample average value of *B*, Cov(*A*,*B*) represents the covariance of *A* and *B*.

(1) $$s_{A,B}=\frac{Cov\left(A,B\right)}{\sigma_{A}\sigma_{B}}\;=\frac{\sum_{i=1}^{n}\left(A_{i}-\overset{-}{A}\right)\left(B_{i}-\overset{-}{B}\right)}{\sqrt{\sum_{i=1}^{n}{\left(A_{i}-\overset{-}{A}\right)}^{2}}\sqrt{\sum_{i=1}^{n}{\left(B_{i}-\overset{-}{B}\right)}^{2}}}$$

The value of *S* is set between −1 and 1. A positive correlation is stronger when its value is closer to 1, while a negative correlation is stronger when its value is closer to −1 [24]. Table 2 shows the value and correlation strength.

### 2.3. Cyclic Neural Network Model

**Figure 2:**
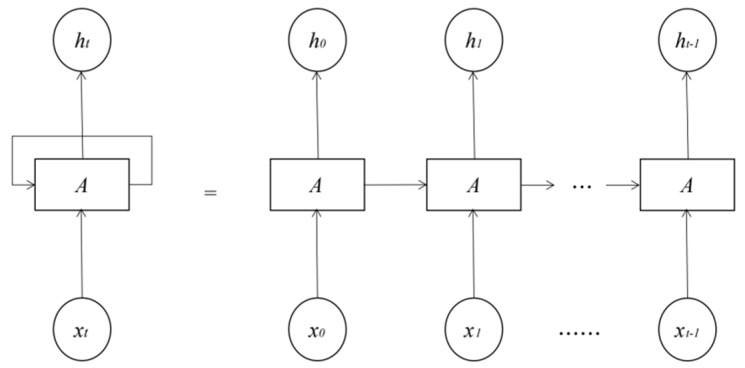
The schematic diagram of neurons and unfolding time series of cyclic neural network.

A recurrent neural network (RNN) is a special network that sorts neurons in a specific order. One of its main functions is to learn and predict the data with time series characteristics. In fact, the structure of the recurrent neural network has only one neuron responsible for the input and output of all data. The data will pass through the output layer, then the middle layer, and finally the output data through the output layer will continue to be input to itself. In this cycle, each cycle is called a frame. Because neurons will bring the information of the previous frame to their next frame, the network is represented by the time axis. The cyclic neural network is expanded according to the time series as a network composed of multiple neurons arranged in sequence, as shown in Figure 2.

**Figure 3:**
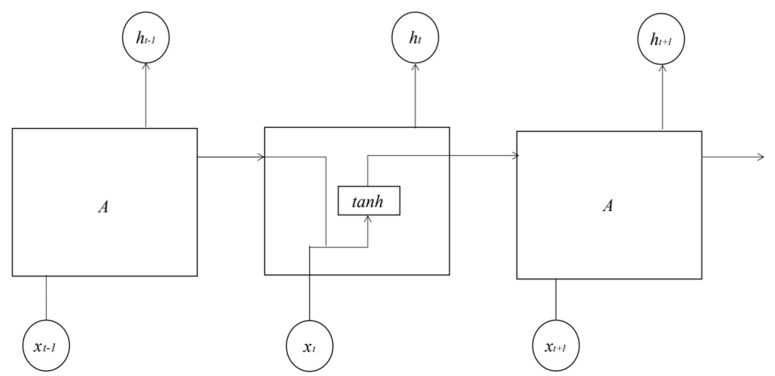
The internal structure diagram of neurons of cyclic neural network.

Figure 3 shows the internal structure diagram of neurons of cyclic neural network. After the expansion of the cyclic neural network, each neuron receives the data transmitted by the neurons in the previous frame as input $X_{\left(t\right)}$, and the calculated value $h_{\left(t\right)}$ will be output to the neurons in the next frame. The input and output are subject to their corresponding weights $\;w_{x\;}$ and restrictions $\;w_{y}$, and the expression is as follows. In Formulas (2) and (3), where $\;h_{\left(t\right)}\;$ is a matrix of m × n, including the output of a small batch of instances at time *t*, *m* is the number of small batch instances and *n* is the number of neurons [25]. $x_{\left(t\right)}\;$ is an *m* × *n* matrix containing all instance inputs. $W_{x}\;$ is an *n* × *n* matrix containing the connection weight of the current time step for each input. $W_{y}\;$ is an *n* × *n* matrix containing the connection weight of the previous time step. b is a bias term of size *N* containing each neuron.

(2) $$h_{\left(t\right)}=\emptyset \left(X_{\left(t\right)}^{T}*w_{x}+y_{\left(t-1\right)}^{T}*w_{y}+b\right)$$

On the last batch of examples, the output of a layer of recursive neurons can be calculated according to the above formula. The expression is as follows:

(3) $$h_{\left(t\right)}=\emptyset \left(X_{\left(t\right)}*W_{x}+Y_{\left(t-1\right)}*w_{y}+b\right)\;=\emptyset \left(\left[X_{\left(t\right)}Y_{\left(t-1\right)}\right]*w+b\right)$$

Notice that $Y_{\left(t\right)}$ is a function of $X_{\left(t\right)}$ and $Y_{\left(t-1\right)}$, $Y_{\left(t-1\right)}$ is a function of $X_{\left(t-1\right)}$ and $Y_{\left(t-2\right)}$, and so on. In this case, $Y_{\left(t\right)}\;$ can be regarded as a function of all inputs $\left(X_{\left(0\right)},X_{\left(1\right)}\right),\ldots ,X_{\left(t\right)}$ from time *t* = 0. In the first step of time, when *t* = 0, there is no previous input, so all previous inputs are initialized to 0.

### 2.4. Gated Recurrent Neural Network

**Figure 4:**
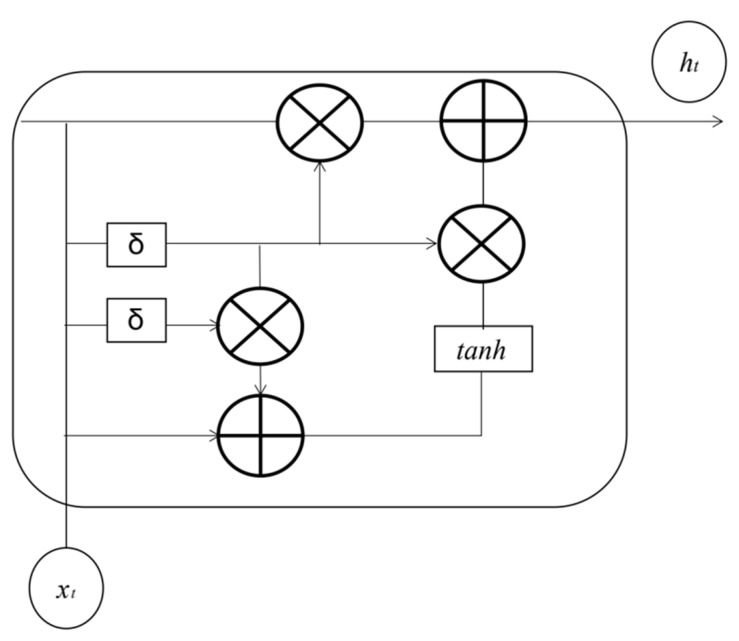
GRU structure.

A gated recurrent neural network (GRNN) is one of the most widely used variants of RNN. As with the long- and short-term memory network, it is also proposed to solve the gradient problem in long-term memory and back-propagation. GRU (gated recurrent unit) has made improvements on the basis of LSTM. The original three door structure of LSTM is retained as only two doors, that is, the input gate and the forgetting gate in LSTM are combined into one to become the update gate. The structure of the GRU model is shown in Figure 4.

GRU combines the internal state vector and output vector into a unified state vector h, and reduces the number of gates to 2: reset gate and update gate. The principle of reset door and update door is as the following Equations (4)–(6) [26].

The reset gate controls the amount that the state $\;h_{t-1}\;$ of the last timestamp enters the GRU. The gating vector $g_{r}$ is obtained by transforming the current timestamp input $x_{t}$ and the previous timestamp state $h_{t-1}$. The specific expression is as follows:

(4) $$g_{r}=\sigma \left(w_{r}\left[h_{t-1},x_{t}\right]+b_{r}\right)$$

where $\;w_{r\;}$ and $\;b_{r\;}$ are the parameters of the reset gate, which are automatically optimized by the back propagation algorithm, and  σ  is the activation function. Generally, sigmoid function is used. When gating vector $g_{r}=0$, the new input ${\tilde{h}}_{t}$ is all from input $x_{t}$, and $h_{t-1}$ is not accepted. At this time, it is equivalent to resetting $h_{t-1}$. When $g_{r}=1$, the $h_{t-1}\;$ and $\;x_{t}\;$ inputs together which will produce a new input ${\tilde{h}}_{t}$.

The gate is updated to control the influence of the last timestamp state $h_{t-1\;}$ and the new input ${\tilde{h}}_{t}$ on the new state vector $h_{t}$. The specific expression is as follows:

(5) $$g_{z}=\sigma \left(w_{z}\left[h_{t-1},x_{t}\right]+b_{z}\right)$$

where $w_{z}$ and $b_{z}$ are the parameters of the update gate, which are automatically optimized by the back propagation algorithm, and  σ  is the activation function. Generally, sigmoid function is used. $g_{z}$ is used to control the new input ${\tilde{h}}_{t}$ signal, and 1 − $g_{z}\;$ is used to control the status $h_{t-1}$ signal:

(6) $$h_{t}=\left(1-g_{z}\right)h_{t-1}+g_{z}{\tilde{h}}_{t}$$

The update quantities of $\;h_{t-1}\;$ and ${\tilde{h}}_{t}$ are in a competitive state. When update gate $g_{z}$ = 0, $h_{t}$ is all from the last timestamp state $h_{t-1}$, and when update gate $g_{z}$ = 1, $h_{t}\;$ is all from the new input ${\tilde{h}}_{t}$.

### 2.5. Bi-Directional Gated Recurrent Neural Network

**Figure 5:**
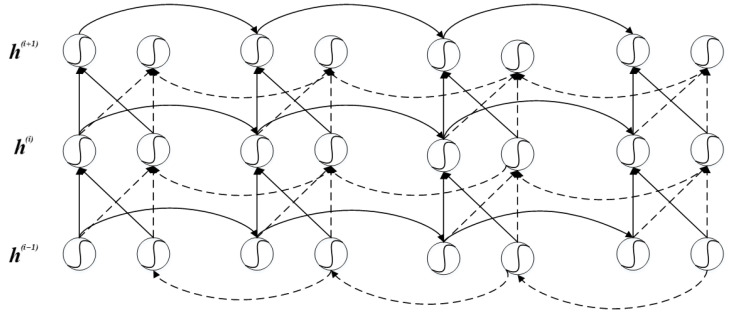
Bi-GRU structure.

Bi-directional gated recurrent neural network is an improved GRU neural network model. The Bi-GRU structure model is shown in Figure 5. A circle represents a GRU unit, and the curve in the circle represents the activation function. A forward propagating GRU unit and a backward propagating GRU unit form a basic unit of Bi-GRU, and several pairs of GRU units form a Bi-GRU deep learning network.

At any time, the input of GRU unit of the current hidden layer has two sources, ${\vec{h_{t-1}}}^{\left(i\right)}$ is the information transmitted from the previous hidden layer, and the other is the information $h_{t}^{\left(i-1\right)}=\left[{\vec{h_{t}}}^{\left(i-1\right)};{\overset{\leftarrow }{h_{t}}}^{\left(i-1\right)}\right]$ transmitted from the previous hidden layer at the current time, including the information transmitted from the front and back directions. The expression corresponding to the information propagated forward $\;{\vec{h_{t}}}^{\left(i\right)}$ and backward ${\overset{\leftarrow }{h_{t}}}^{\left(i\right)}\;$ of the *i*th hidden layer is as follows:

(7) $${\vec{h_{t}}}^{\left(i\right)}=f\left({\vec{W}}^{\left(i\right)}h_{t}^{\left(i-1\right)}+{\vec{V}}^{\left(i\right)}{\vec{h_{t-1}}}^{\left(i\right)}+{\vec{b}}^{\left(i\right)}\right)$$

(8) $${\overset{\leftarrow }{h_{t}}}^{\left(i\right)}=f\left({\overset{\leftarrow }{W}}^{\left(i\right)}h_{t}^{\left(i-1\right)}+{\overset{\leftarrow }{V}}^{\left(i\right)}{\overset{\leftarrow }{h_{t+1}}}^{\left(i\right)}+{\overset{\leftarrow }{b}}^{\left(i\right)}\right)$$

where: f is the activation function; ${\vec{W}}^{\left(i\right)}$, ${\vec{V}}^{\left(i\right)}$ and ${\vec{b}}^{\left(i\right)}$ respectively represent the two forward weights and offsets of layer I; ${\overset{\leftarrow }{W}}^{\left(i\right)}$, ${\overset{\leftarrow }{\;V}}^{\left(i\right)\;},$ and ${\overset{\leftarrow }{b}}^{\left(i\right)}$ represent the two backward weights and offsets of layer I, respectively.

### 2.6. Experimental Design

As various air quality monitoring stations may encounter many situations such as equipment abnormality when collecting data, the collected data may have blank values. Here, because the data vacancy value used in the experiment accounts for a relatively small proportion, but considering that the change in particle concentration is highly dependent, direct deletion will damage the integrity of the data, so the linear interpolation method is used to fill the vacancy value. As the change in particle concentration has obvious seasonal characteristics, the environmental pollution impact field of the seasonal type is added.

The experimental data contain 13 environmental pollution impact factors, and the meanings of each field are shown in Table 3.

When training the model, the data type needs to be converted into numerical type. Because the experimental data contain character type data, namely rain and season fields, classification data processing is required. The specific operation is to convert characters representing different meanings into different numerical values.

Considering that the dimensions of data fields are inconsistent, dimensionless operation is required. Generally, there are two methods for dimensionless processing, namely, normalization and standardization. However, because there are some outliers and noises in the data, the standardized processing can indirectly avoid the impact caused by outliers. Therefore, standardized processing is adopted here. The standardized calculation formula is as follows:

(9) $$x^{*}=\frac{x_{i}-\overset{-}{x}}{\sigma }$$

where $x_{i}$ and $x^{*}$ respectively represent the original observation value and the normalized observation value of each field. Additionally, they respectively represent the mean and standard deviation of all observed values.

Here, feature engineering is divided into two parts, one is the construction of data sets, the other is the division of data sets. First, we constructed the data set, and then we divided the data into two parts. One part is the data containing only PM_2.5_, and the other part is the data containing all features. The data set is divided into training set and test set. The proportion of this paper is divided according to 8:2, that is, 80% of the data is used as training set for model training, and 20% of the data is used as test set to evaluate the performance of the model.

In order to evaluate the performance of model prediction, this paper used two different indicators, including mean absolute error (MAE) and root mean square error (RMSE). The calculation methods of these two indicators are as follows.

(10) $$MAE=\frac{1}{N}\sum_{1}^{N}{\left({observed}_{t}-{predicted}_{t}\right)}^{2}$$

(11) $$RMSE=\sqrt{\frac{1}{N}\sum_{1}^{N}{\left({observed}_{t}-{predicted}_{t}\right)}^{2}}$$

where *observed_t_* represents the observed value at time *t* and *predicted_t_* represents the predicted value at time *t*.

**Figure 6:**
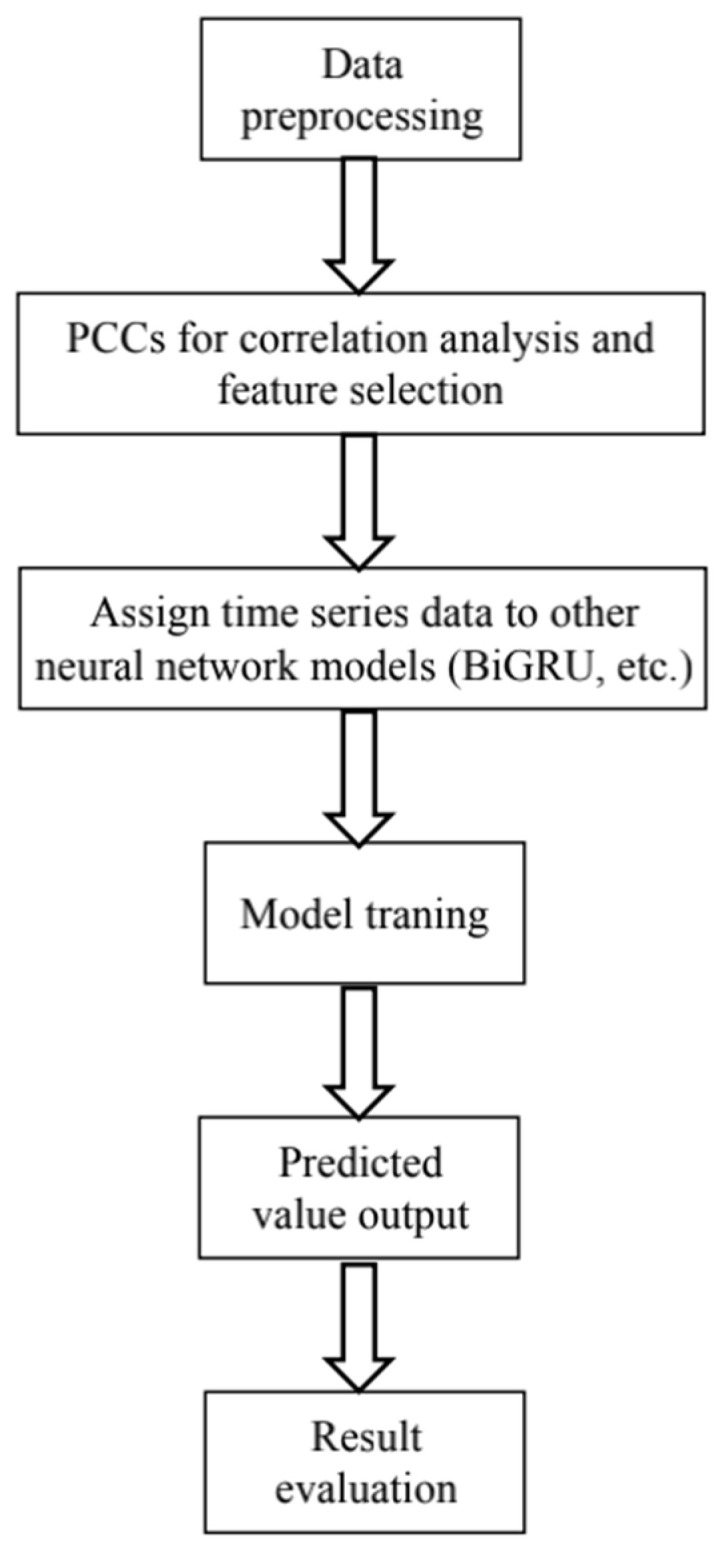
The flow chart of prediction based on PCCs and Bi-GRU neural network.

**Figure 7:**
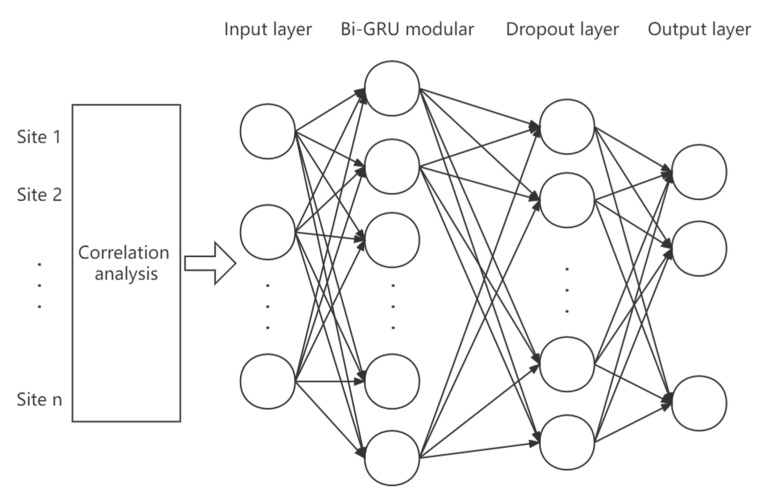
The structure diagram of neural network model based on PCCs-BiGRU.

The flow chart of the PCCs-BiGRU network for air pollutant concentration prediction is shown in Figure 6, and the model structure is shown in Figure 7.

In order to analyze the impact of space adjacent sites on the change of air pollutant concentration at the central site, the correlation analysis proposed above was used to screen the target pollutant concentration at the adjacent sites, and then the screened features and the historical data of the central site were assigned to the Bi-GRU model as inputs to obtain the final prediction results.

The initialization values of the network structure parameters are as follows:

1. Input and output vector dimensions: the input contains a total of 16 pollutant concentrations including PM_2.5_ at each time before prediction and related impact factors, and the output is the predicted PM_2.5_ concentration in the next six hours. Since the time frequency of the data is hours, the time step set by the model here is 24, that is, the PM_2.5_ concentration in the next six hours is predicted by using the 24-h historical air pollution related data. The number of neurons in the Bi-GRU module of the model is set to 64. In order to prevent over fitting, the dropout is set to 0.2, that is, 20% of the output is randomly screened. The final output layer of the network is the sense layer with a dimension of six, which represents the concentration of PM_2.5_ in the six hours after prediction.
2. Loss function: the loss function of the neural network uses the MAE (mean absolute error) between the predicted PM_2.5_ value and the real value to make the predicted PM_2.5_ concentration output by the network as close to the real value as possible.
3. Optimization method: the Adam optimizer is used here. Through a large number of theories and practices, it has been proven that the Adam optimization method has better performance than other adaptive learning methods. The Adam convergence speed is faster and it is more suitable for processing sparse data.

**Figure 8:**
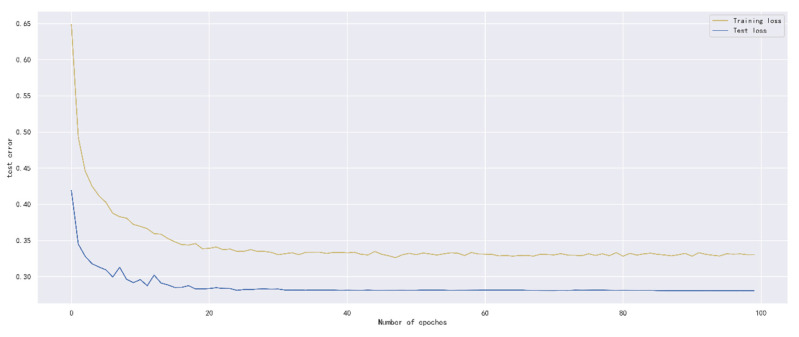
The relationship between Bi-GRU model and training rounds.

Next, we trained the model with the prediction step of six hours, and compared the convergence speed of the model and the experience accumulated by the prediction error through experiments. In the training rounds, epochs were set to 100, and the number of training samples in each batch size was set to 64. The error performance of the model during training and testing is shown in Figure 8.

It is concluded from the curve that after the training rounds of the model reach 40, the error change of the training set is no longer obvious, and basically tends to be stable. In order to speed up the training speed on the premise of reducing errors, it is reasonable to set the rounds to 50.

## 3. Experimental Results and Analysis

### 3.1. Experimental Results of PM_2.5_ Concentration Prediction Based on PCCs and Bi-GRU

**Figure 9:**
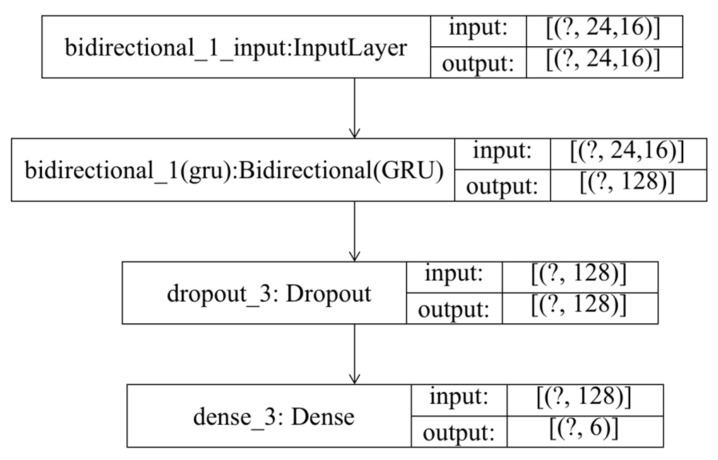
Schematic diagram of PCCs-BiGRU structure model.

In this experiment, the time step set by Bi-GRU is 24 and the number of neurons is 64. The optimizer uses Adam. In order to prevent over fitting, the dropout layer is added and set to 0.2, and the parameter of the sense layer is set to six, that is, the PM_2.5_ concentration in the next six hours is predicted. Here, the training set and test set are divided by 8:2, that is, the training set is set as the first 1168 groups and the test set as the last 292 groups. The structure of the experimental model is shown in Figure 9.

Since the target of our prediction here is the PM_2.5_ concentration of the Olympic Sports Center site, the PM_2.5_ concentration characteristics of the three sites with the strongest correlation with the PM_2.5_ concentration characteristics of the target site are screened through PCCs, and the corresponding correlation table is obtained through experiments, as shown in Table 4. If more sites of related data are selected, there will be too many influence fields in the final fused data set, which will lead to slowing the speed of the model and a long training time, which will affect the model accuracy and efficiency. Through the simulation experiments, the three sites with the highest correlation are selected to ensure high accuracy in a certain extent, and that the complexity of the training model will not be too high.

It can be seen from Table 4 that the three adjacent stations with the strongest correlation with the PM_2.5_ concentration of the target station are Guanyuan, Dongsi, and the agricultural exhibition hall. Therefore, the PM_2.5_ concentration of the three stations and the historical pollutant concentration of the central station are added to the model for training as inputs during the model training.

**Figure 10:**
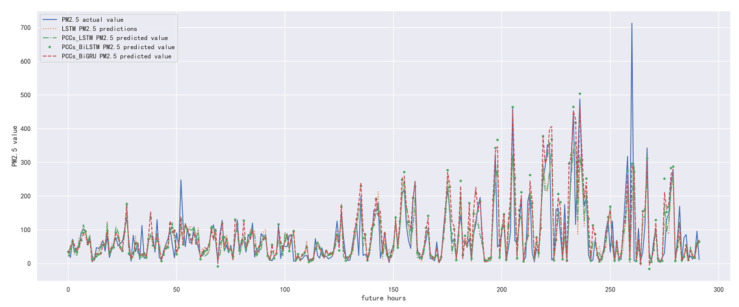
The schematic diagram of prediction results of PM_2.5_ concentration in the next one hour.

**Figure 11:**
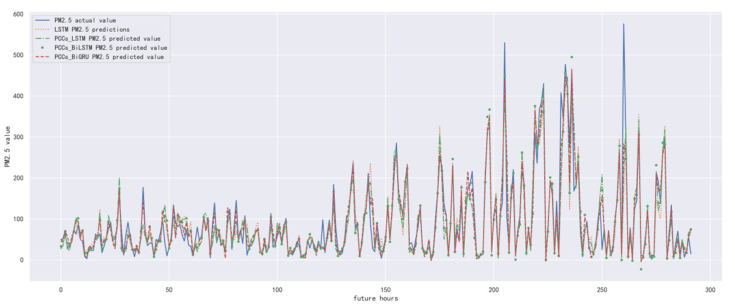
The schematic diagram of predicted PM_2.5_ concentration in the next two hours.

**Figure 12:**
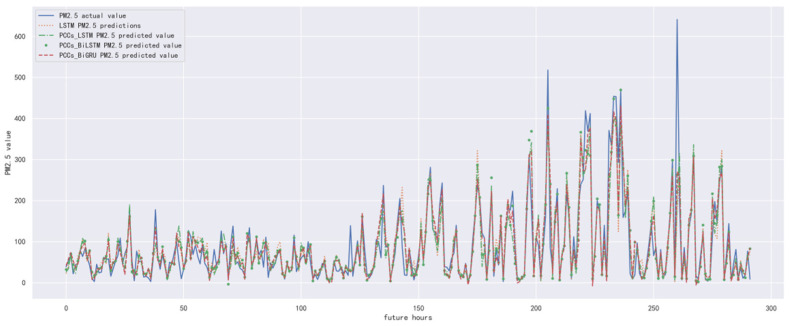
The schematic diagram of predicted PM_2.5_ concentration in the next three hours.

**Figure 13:**
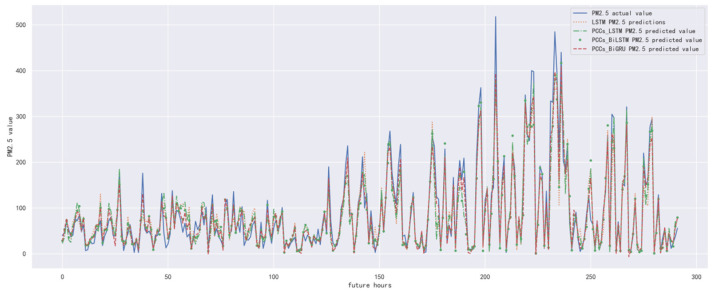
The schematic diagram of predicted PM_2.5_ concentration in the next four hours.

**Figure 14:**
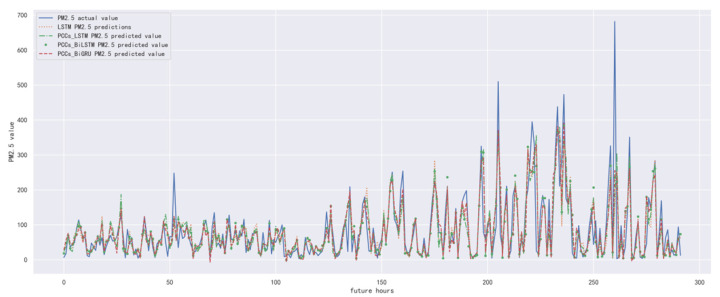
The schematic diagram of prediction results of PM_2.5_ concentration in the next five hours.

**Figure 15:**
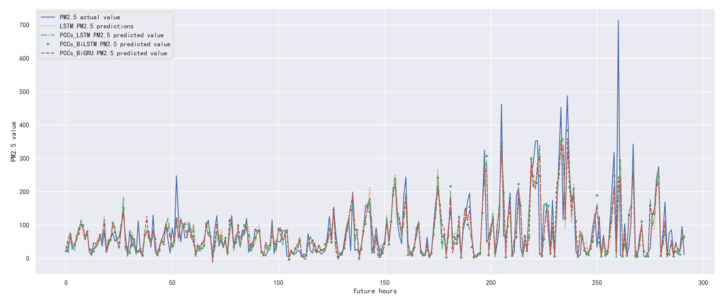
The schematic diagram of predicted PM_2.5_ concentration in the next six hours.

In order to compare the performance of correlation analysis and Bi-GRU prediction models, we compared them with other algorithms, including LSTM prediction model without correlation analysis, correlation analysis and LSTM prediction model, and correlation analysis and Bi-LSTM prediction model. The predicted results of the PM_2.5_ concentration corresponding to each model in the test set in the next six hours are shown in Figure 10, Figure 11, Figure 12, Figure 13, Figure 14 and Figure 15, and the corresponding error indicators of the predicted results of each model are shown in Table 5 and Table 6.

It can be seen from Table 5 and Table 6 that the MAE average and RMSE average of the four models decrease in turn, indicating that the performance of the model is improving after the introduction of correlation analysis and the BiGRU model. Similarly, we also observe that the prediction error increases with the increase in prediction duration because this is a normal phenomenon caused by error accumulation.

### 3.2. Experimental Results of PM_10_ Concentration Prediction Based on PCCs and Bi-GRU

Since the target of our prediction here is the PM_10_ concentration of the Olympic Sports Center site, we first screen the PM_10_ concentration characteristics of the first three sites with the strongest correlation with the PM_10_ concentration characteristics of the target site through PCCs, and the corresponding correlation table is shown in Table 7 through experiments.

It can be seen from Table 7 that the three adjacent stations with the strongest correlation with the PM_10_ concentration of the target station are Dongsi, Guanyuan, and agricultural exhibition hall. It is noted that the three adjacent stations with the strongest correlation with the PM_2.5_ concentration characteristics of the target station are the same. In environmental science, the concentration changes in PM_2.5_ and PM_10_ are positively correlated, and the experimental results here also prove this. Therefore, during model training, the PM_10_ concentration of the three stations and the historical pollutant concentration of the central station are added to the model as inputs for training.

**Figure 16:**
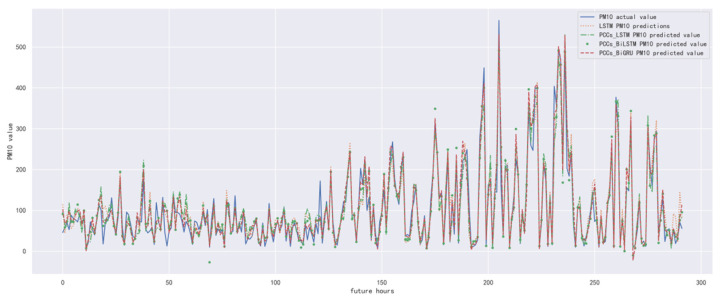
The schematic diagram of predicted PM_10_ concentration in the next one hour.

**Figure 17:**
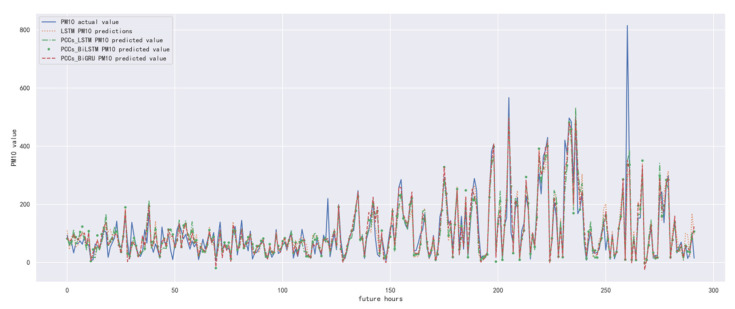
The schematic diagram of predicted PM_10_ concentration in the next two hours.

**Figure 18:**
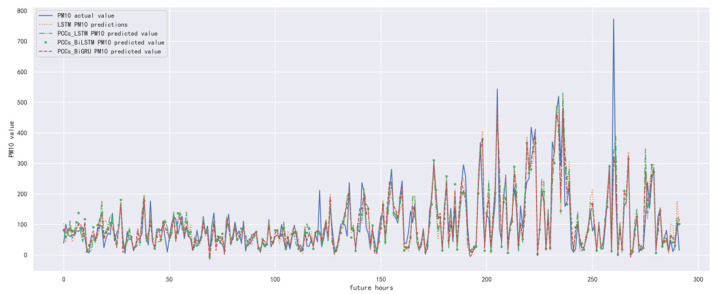
The schematic diagram of predicted PM_10_ concentration in the next three hours.

**Figure 19:**
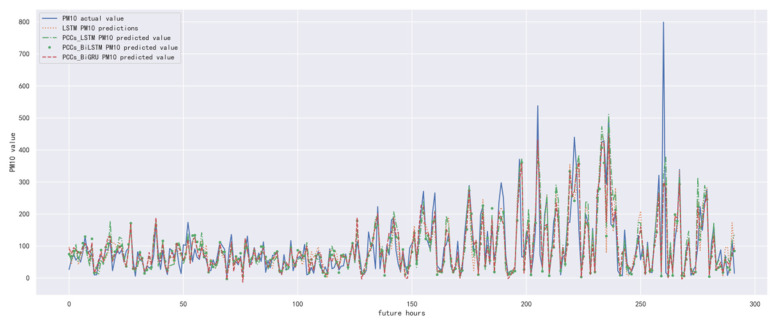
The schematic diagram of predicted PM_10_ concentration in the next four hours.

**Figure 20:**
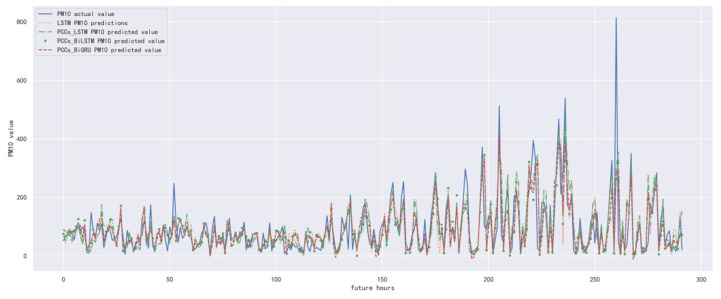
The schematic diagram of predicted PM_10_ concentration in the next five hours.

**Figure 21:**
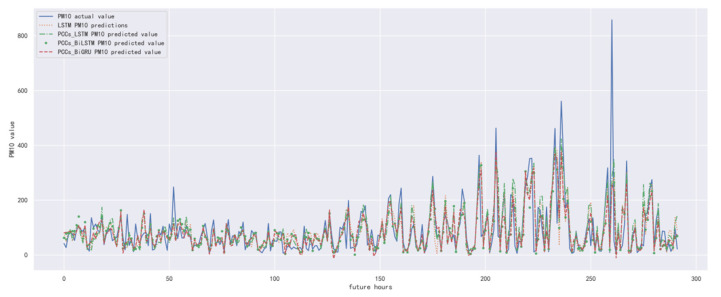
The schematic diagram of predicted PM_10_ concentration in the next six hours.

In order to compare the performance of the correlation analysis and Bi-GRU prediction models, we compared them with other algorithms, including the LSTM prediction model without correlation analysis, the correlation analysis and LSTM prediction models, and the correlation analysis and Bi-LSTM prediction models. The predicted results of PM_10_ concentration corresponding to each model in the test set in the next six hours are shown in Figure 16, Figure 17, Figure 18, Figure 19, Figure 20 and Figure 21, and the corresponding error indicators of the predicted results of each model are shown in Table 8 and Table 9.

It can be seen from Table 8 and Table 9 that the MAE average and RMSE average of the four models decrease in turn, indicating that the performance of the model is improving after the introduction of correlation analysis and the BiGRU model.

## 4. Discussion

From the experimental results, we can make the comparison of MAE and RMSE for the prediction results of our method in the next six hours with the other three prediction models. First, we can see that the worst prediction effect in the first three hours is the LSTM prediction model without correlation analysis, which is the most commonly used method for particle concentration prediction, that is, the prediction model obtained by directly training the target site data set in the LSTM model. Second, the PCCs-LSTM prediction model has a better effect, which is significantly improved compared with the previous LSTM prediction model without correlation analysis. The reason is obviously that the correlation analysis is carried out, and the data set of the target site and the data set of several sites with the strongest correlation are combined and then put into the model for training, so the prediction effect is improved. The prediction effect of the PCCs-BiLSTM model is better than that of the PCCs-LSTM model. This is because the LSTM model itself is better at capturing the information of the far position than that of the near position, and the BiLSTM model avoids this problem. It can be seen that the PCCs-BiGRU model proposed in this paper has the best prediction effect compared with the other three comparison models. On the one hand, it selects the training data set after correlation analysis, which can better mine the internal relationship between the data of the target site and the data of the target site. This is also the key part of the excellent prediction effect of the model. Second, the BiGRU model is used for model training, because compared with BiLSTM, BiGRU is equivalent to simplified operation, which can save more hardware computing power and time cost, and the prediction effect will be better.

We also see that the prediction effect of the PCCs-LSTM model has not been improved compared with the LSTM prediction model without correlation analysis in the last three hours, because of the disadvantages of the LSTM model. The PCCs-BiLSTM and PCCs-BiGRU models do not have similar situations, and the prediction effect of PCCs-BiGRU obtains the best results in most conditions. In conclusion, the PCCs-BiGRU prediction model proposed in this paper is more effective in predicting the change in particle concentration.

However, there is a significant difference between the predicted results and the actual results of the fine particle concentration at the target station at some time, which is caused by various factors. We can see from the results that when the difference is relatively large, it is often far greater than the fine particle concentration in the adjacent period. We often refer to the data that deviates from the average value in the time series data set as outliers. There are many reasons for this situation, such as the error caused by the failure of the monitoring station during data sampling, and the data obtained by the automatic completion method corresponding to the loss of data at a certain time. The outliers will have impact on the future time series analysis. The outliers will directly affect the fitting accuracy of the model, so there will cause a large difference between the predicted results and the actual values at a certain time. For this situation, we can reduce it as much as possible during data preprocessing, but we cannot completely avoid it. Considering the occurrence of this situation, we also conducted averaging processing for the selection of the two evaluation index results of MAE and RMSE in this paper, which can reflect the advantages and disadvantages of our overall prediction in different time.

Because the value of the Pearson coefficient ranges from −1 to 1, and a positive correlation is stronger when its value is closer to 1, while a negative correlation is stronger when its value is closer to -1, in this paper, we selected the three sites with the highest correlation with the change in fine particles at the target site, so the corresponding Pearson coefficient, whether it is close to 1 or −1, is considered to be highly correlated. Therefore, we treat the absolute value of its Pearson coefficient, so its range is 0 to 1, shown in Table 2.

In addition, the method proposed in this paper also has some disadvantages. For example, regarding the data source, we need to obtain as much data as possible from adjacent sites to facilitate the correlation analysis among sites, so the requirements for data source acquisition are relatively high. In the experiments, we predicted the concentration of fine particles in the next six hours, and the overall prediction effect is good. If the method is used for longer time prediction, the results are not good. The advantage is that our method can perform real-time and accurate forecasting particulate concentration at the hour level during the next six hours.

## 5. Conclusions

In this study, we first conducted the correlation analysis of fine particle concentration at the target site through PCCs, then combined it with the Bi-GRU model and established a new prediction model which is used for the regional prediction of fine particle concentration in the air. This model can fully consider the internal relationship among the air quality monitoring stations by combining the influence of the correlation among the stations. The experiment used the hourly PM_2.5_ and PM_10_ fine particle concentration data of 12 air quality monitoring stations in Beijing, and selected the Olympic Sports Center Station as the target station, and the other 11 stations as the adjacent stations. The proposed prediction model of fine particle concentration based on correlation analysis and Bi-GRU was trained, verified, and tested. The experimental results show that: (1) In the first three hours, the prediction error of PCCs-LSTM prediction model was smaller than that of LSTM prediction model without correlation analysis. (2) The prediction error between the PCCs-LSTM prediction model and the LSTM prediction model of correlation analysis in the last three hours is almost showing no big difference. (3) In the first six hours, the prediction error of PCCs-BiLSTM prediction model is smaller than that of PCCs LSTM prediction model. (4) The prediction error of the PCCs-BiGRU prediction model proposed in this paper within the first six hours is smaller than that of the other three prediction models. (5) The prediction error of each model increases with the increase in prediction time step.

In terms of application, the model can help environmental researchers to further improve the prediction accuracy of the concentration of fine particles in the air, and provide tools for environmental policy makers to implement relevant pollution control policies. Through the correlation analysis between the target site and the adjacent sites, the internal relationship can be mined to provide decision support for accurate pollution control. The limitation of this study is that only the data sets in Beijing were selected and only the fine particle concentration prediction in the next six hours were carried out. In future research, we can consider selecting data sets from more regions and predicting fine particle concentration in a longer time step to further verify the superiority of the PCCs-BiGRU prediction model proposed in this paper in fine particle concentration prediction.

## Figures and Tables

**Table 1:** Station longitude and latitude coordinates.

No.	Site Name	Longitude	Latitude
(1)	Olympic Sports Center	116.403458	39.989664
(2)	Changping	116.233032	40.229940
(3)	Dingling	116.232467	40.300231
(4)	Dongsi	116.435012	39.938650
(5)	Guanyuan	116.369366	39.937364
(6)	Gucheng	116.195640	39.913450
(7)	Huailou	116.646984	40.308425
(8)	Agricultural Exhibition Hall	116.470307	39.947009
(9)	Shunyi	116.664263	40.177008
(10)	Temple of Heaven	116.417313	39.887977
(11)	Wanliu	116.305661	39.972746
(12)	Longevity West Palace	116.374728	39.885653

**Table 2:** Classification of correlation strength.

Pearson Coefficient Value Range (Absolute Value)	Strength Level
(0.8, 1.0]	Extremely strong correlation
(0.6, 0.8]	Strong correlation
(0.4, 0.6]	Closely correlation
(0.2, 0.4]	Weak correlation
[0.0, 0.2]	Irrelevance

**Table 3:** The meanings of data fields.

Field Name	Representative Meaning	Unit
PM_2.5_	PM_2.5_ Concentration	(µg/m^3^)
PM_10_	PM_10_ Concentration	(µg/m^3^)
SO_2_	SO_2_ Concentration	(µg/m^3^)
NO_2_	NO_2_ Concentration	(µg/m^3^)
CO	CO Concentration	(µg/m^3^)
O_3_	O_3_ Concentration	(µg/m^3^)
TEMP	Temperature	°C
PRES	Pressure	Hpa
DEWP	Dew Point Temperature	°C
RAIN	Precipitation	mm
wd	Wind Drection	/
WSPW	Wind Speed	m/s
season	Season	/

**Table 4:** PM_2.5_ correlation of each station.

Site Name	PCCs
Olympic Sports Center	1.000
Guanyuan	0.954
Dongsi	0.949
Agricultural Exhibition Hall	0.943
Wanliu	0.940
Temple of Heaven	0.930
Longevity West Palace	0.920
Gucheng	0.900
Shunyi	0.891
Huailou	0.847
Changping	0.841
Dingling	0.821

**Table 5:** The MAE of PM_2.5_ concentration prediction in the next six hours.

Model Name	LSTM	PCCs-LSTM	PCCs-BiLSTM	PCCs-BiGRU
Next 1 h	21.07	17.79	13.47	11.18
Next 2 h	24.72	22.24	18.42	16.63
Next 3 h	27.69	26.51	23.17	21.07
Next 4 h	30.07	29.55	26.46	25.50
Next 5 h	32.29	32.40	30.37	29.09
Next 6 h	33.79	33.74	32.42	31.38

**Table 6:** The RMSE of PM_2.5_ concentration prediction in the next six hours.

Model Name	LSTM	PCCs-LSTM	PCCs-BiLSTM	PCCs-BiGRU
Next 1 h	31.95	27.97	20.40	19.05
Next 2 h	44.19	40.57	33.03	32.13
Next 3 h	48.64	46.99	41.52	39.11
Next 4 h	54.32	53.94	49.24	47.98
Next 5 h	58.49	59.61	54.11	53.04
Next 6 h	61.91	62.09	58.55	56.48

**Table 7:** PM_10_ correlation of each station.

Site Name	PCCs
Olympic Sports Center	1.000
Dongsi	0.918
Guanyuan	0.917
Agricultural Exhibition Hall	0.912
Wanliu	0.902
Temple of Heaven	0.882
Longevity West Palace	0.880
Shunyi	0.855
Gucheng	0.845
Changping	0.808
Huailou	0.782
Dingling	0.770

**Table 8:** The MAE of PM_10_ concentration prediction in the next six hours.

Model Name	LSTM	PCCs-LSTM	PCCs-BiLSTM	PCCs-BiGRU
Next one hour	22.56	21.24	18.31	15.95
Next two hours	28.22	27.40	25.25	22.90
Next three hours	32.73	31.94	29.47	26.15
Next four hours	34.08	36.72	32.82	30.80
Next five hours	37.14	40.42	36.97	36.20
Next six hours	38.71	41.09	38.33	37.10

**Table 9:** The RMSE of PM_10_ concentration prediction in the next six hours.

Model Name	LSTM	PCCs-LSTM	PCCs-BiLSTM	PCCs-BiGRU
Next one hour	32.78	30.78	26.75	23.73
Next two hours	48.81	47.19	44.21	42.21
Next three hours	53.89	52.97	49.43	45.18
Next four hours	59.39	63.37	57.94	54.59
Next five hours	63.12	68.50	62.27	61.00
Next six hours	67.30	71.20	66.94	64.65

## Data Availability

The data presented in this study are available from the corresponding author on reasonable request.
